# Pilot randomized trial on mindfulness training for smokers in young adult binge drinkers

**DOI:** 10.1186/1472-6882-13-215

**Published:** 2013-09-03

**Authors:** James M Davis, David M Mills, Kristin A Stankevitz, Alison R Manley, Matthew R Majeskie, Stevens S Smith

**Affiliations:** 1 Center for Tobacco Research and Intervention, University of Wisconsin School of Medicine and Public Health, 1930 Monroe Street, Suite 200, 53711 Madison, WI, USA; 2 Department of Medicine, University of Wisconsin School of Medicine and Public Health, 1685 Highland Avenue, 53705 Madison, WI, USA

**Keywords:** Smoking, Tobacco, Alcohol, Mindfulness, Young adult

## Abstract

**Background:**

We report results of a pilot study designed to test a novel smoking cessation intervention, Mindfulness Training for Smokers (MTS), in smokers age 18-29 years with regular episodes of binge drinking. Mindfulness is a cognitive skill of applying close moment-to-moment attention to experience with a mental posture of acceptance and non-reactivity. The MTS intervention consisted of six weekly classes that provided instruction on how to use mindfulness to manage known precursors of smoking relapse including smoking triggers, strong emotions, stressful situations, addictive thoughts, urges, and withdrawal symptoms.

**Methods:**

The MTS intervention was compared to Interactive Learning for Smokers (ILS), a time/intensity matched control group using daily non-directed walking instead of mindfulness meditation. Recruitment was conducted primarily at local technical colleges. Primary outcome measures included biochemically-confirmed smoking abstinence and reduction in alcohol use at the end of treatment (2-weeks post-quit attempt).

**Results:**

The sample (N = 55) was 70.9% male, with a mean age of 21.9 years, and a mean of 11.76 alcoholic drinks consumed per week. Intent-to-treat analysis showed biochemically-confirmed 7-day point prevalence abstinence rates at 2-weeks post-quit for MTS = 20.0% and ILS = 4.0%, *p* = .08. Secondary analysis showed number of drinks per week in the first 2-weeks post-quit correlated with smoking relapse at 2-weeks post-quit (*p* < .01).

**Conclusions:**

This pilot study demonstrated that Mindfulness Training for Smokers shows promise for smoking cessation and alcohol use reduction in treating young adult smokers with alcohol abuse. Results suggest the need for a study with larger sample size and methods that reduce attrition.

**Trial registration:**

ClnicalTrial.gov, NCT01679236

## Background

Smoking is the leading preventable cause of morbidity and mortality in the United States, causing 443,000 deaths and national costs of $96 billion each year [1], with 23.1% of men and 18.1% of women self-identified as cigarette smokers [2]. Smoking rates are highest in young adults, with a 2010 survey showing that 35.8% of 21–25 year old US adults smoked at least once in the past 30 days [3]. Young adults are uniquely susceptible to social and peer group influences leading to substance use and smoking [4]. The majority of smokers (88%) begin smoking before the age of 18, and 99% of smokers begin smoking before the age of 26 [5]. Tobacco companies take advantage of the experimental processes inherent in young adult development by targeting young people through advertising [6-8]. Many of the chronic diseases associated with smoking are more common among those who start smoking early in life [9-11], and smoking cessation early in life provides the greatest benefit in terms of reduced morbidity and mortality [12]. There has been a considerable effort toward reduction of tobacco use through development of smoking cessation medications [13], behavioral interventions [14], passage of public smoking bans [15], mass media campaigns [16], and government smoking bans [17,18]. Unfortunately, there has been little progress in the development of interventions targeted to young adult smokers [19,20]. Over the last 50 years, there have been only a small number of studies published on smoking interventions targeted to young adults [21]. These include a 1972 study in which male undergraduate smokers were exposed to 24 hours of sensory deprivation as a smoking cessation therapy [22], a 1988 study that compared a brief counseling intervention vs. no intervention [23], a 1990 study that evaluated a behavioral therapy in undergraduate smokers [24], and a 2007 study that applied a brief office intervention vs. the same plus expressive writing in young adult smokers [25]. Currently, the majority of young adult smokers attempt smoking cessation without the help of medications or a smoking cessation program [26] and achieve long-term abstinence rates of less than five percent [27]. A barrier to development of interventions targeting young adult smokers is that this population is known to have high attrition in study protocols and poor adherence to behavioral therapies [28,29]. The prospect of poor study outcomes with this population may lead researchers to study treatments in more compliant adult populations.

Young adult smokers face unique challenges in achieving smoking abstinence and accordingly, smoking cessation programs designed for young smokers must develop unique strategies to overcome those challenges. One of the most widely described challenges to young adult smokers is the high prevalence of binge drinking and the difficulties of maintaining abstinence during binge drinking [3,30-34]. Binge drinking has been defined as consuming four or more alcoholic drinks on one or more occasion for women and five or more drinks on one or more occasion for men [35]. The National Survey on Drug Use and Health (NSDUH) found that 40.6% of young adults, ages 18–25 years, had at least one binge drinking episode in the past 30 days [3]. Alcohol consumption has been shown to increase the intensity of smoking urges [36,37] and predict subsequent smoking urges [38,39]. One study showed that subjects who engaged in binge drinking were more likely to have smoking relapse within the first year and to smoke more cigarettes after relapse occurred [40]. Another study showed that the probability of smoking relapse on heavy drinking days was significantly higher than smoking on moderate drinking or abstinent days [41]. There is now a growing body of evidence to suggest that simultaneous treatment of smoking and alcohol abuse leads to higher rates of abstinence in both [42-45], and there is growing interest in providing treatment for alcohol and tobacco use concurrently [46,47]. Nonetheless, a recent literature review using PubMed and PsycINFO revealed only one published study on the treatment of smoking cessation and binge drinking in young adults. In this study 41 smoking binge drinkers age 18–30 years were randomized to receive either medications and semi-structured smoking cessation counseling, or the same but with a brief alcohol intervention. This study showed promising effect sizes between groups favoring the dual treatment arm, but was not powered to reach statistical significance [46]. Cognitive-behavioral therapies (CBT) and acceptance and commitment therapies (ACT) have shown success in the treatment of alcohol abuse [47,48] and smoking [49]. Recent studies suggest that mindfulness training, which has similarities with ACT, also shows promise for alcohol abuse and smoking [50-53]. Research on Mindfulness Based Relapse Prevention (MBRP) has demonstrated preliminary evidence for efficacy in the treatment of alcohol dependence [54,55]. A pilot study on mindfulness training in smokers found a 56% rate of abstinence at 6-weeks post-quit [56] and a trial comparing a mindfulness-training program to an active control found that mindfulness training yielded significantly higher smoking abstinence at 17-weeks post-quit [57].

The current study was designed to test a mindfulness intervention, “Mindfulness Training for Smokers” (MTS), that was developed as a treatment for smoking cessation. The goal in this study was to teach mindfulness skills targeted to smoking addiction and then see if the mindfulness skills obtained might generalize to decrease alcohol use. The study employed NIH-endorsed research methods for a stage-1 behavioral therapy program development [58] with identified outcomes of exploring treatment acceptability and adherence and establishing initial efficacy estimates necessary to guide future research. MTS was designed to be intensive enough to activate core mindfulness insights of a Mindfulness Based Stress Reduction (MBSR)-style course [59], but also include training on how to use mindfulness to more skillfully manage relapse challenges related to smoking triggers, social situations, strong emotions, stressful situations, relapse-related thoughts, urges and withdrawal symptoms. A similar name “Mindfulness Training for Smoking Cessation” has been used to describe another recently published smoking cessation intervention with different structure developed by Brewer [57]. The two interventions were developed concurrently, and because materials were already in use at the time of Brewer’s publication, we continued to describe this program, as “Mindfulness Training for Smokers.” Early assessment of participant population (students from small towns around the state) revealed that they would be unable to attend long-term follow up visits. For this reason, the study assessed outcomes only at the end-of-treatment, i.e., two weeks after the quit day. Primary outcomes were defined as smoking abstinence confirmed via carbon monoxide (CO) breath testing with daily smoking assessed via timeline followback (TLFB) [60] at two weeks post-quit.

## Methods

### Procedures

**Figure 1:**
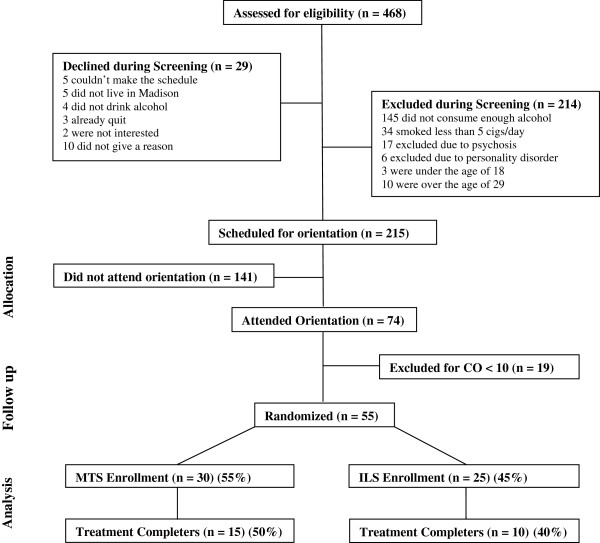
Consort diagram.

Recruitment took place in the Madison, Wisconsin area and was conducted primarily through flyers and “one-minute classroom presentations” at local community colleges. Callers were provided with a brief description of the study, and if interested, underwent phone screening. Inclusion criteria required that participants be 18 to 29 years old, smoke 10 or more cigarettes per day, and report 5 or more alcohol “binges” per month. A binge was defined as 5 or more drinks per day for males and 4 or more drinks for females [35]. Participants were excluded for possible alcohol dependence if they reported drinking 4 or more drinks on 6 or more nights per week. Participants were also excluded if they self-reported a diagnosis of schizophrenia, bipolar or delusional disorder. Those who passed phone screening were invited to attend an orientation session. Those who attended an orientation session were excluded if carbon monoxide (CO) breath testing showed a CO level of 10 parts per million (ppm) or less. The orientation then included a description of the study, after which interested individuals signed the consent form and completed baseline testing. Subjects were then randomized into either Mindfulness Training for Smokers (MTS) or the control condition “Interactive Learning for Smokers” (ILS) by random draws. Descriptions of the two interventions and other processes were provided in such a way so as to decrease the possibility that controls might know that they had been randomized to a control arm. Phone screening was conducted on 468 callers leading to 215 callers scheduled for orientations. The main reason for exclusion was insufficient alcohol use (less than 5 binges per month). Of scheduled callers, 74 (34%) attended an orientation, after which 55 were randomized to MTS (n = 30) or ILS (n = 25). Among these 55 participants, a total of 25 (45%) completed treatment (attended Quit Day Retreat) and testing (two-weeks post-quit assessment visit), including 15 MTS participants and 10 ILS participants (Figure 1). This study was carried out in compliance with the guidelines provided by the Helsinki Declaration, and was approved and overseen by the University of Wisconsin-Madison Health Sciences Internal Review Board (H-2006-0279).

### Measurements

Study assessment visits were performed at baseline and at the end of treatment (two weeks after the Quit Day Retreat). The following measures were used for data collection:

1) *Tobacco and alcohol use*. Two metrics were used to assess post-quit day smoking behavior: a self-report testing via “timeline followback” (TLFB) calendar [60-62], and biomedical testing via carbon monoxide (CO) breath testing. Participants were asked to log cigarette consumption via TLFB each day throughout the intervention until 2 weeks after the quit day. For CO breath testing, the study utilized the Micro + ™ Smokerlyzer® and employed a more stringent CO cutoff of 7 ppm (instead of 10 ppm) to reduce the chance of false positives for a coding of abstinent [63]. Failure to provide self-report of no cigarette consumption during the seven days prior to the post-quit assessment visit, or to provide a CO reading of under 7.0 ppm on the breath test, or to attend an assessment visit all resulted in coding as “relapsed”. A contemporary measure “number of days smoked in the first two weeks post-quit” has been shown to correlate well with 6-month smoking cessation outcomes [64], and was used to provide a continuous measure of smoking abstinence. Daily alcohol use was also assessed using TLFB, wherein participants recorded the number of alcoholic drinks that they consumed each day throughout the intervention until two weeks after the quit day. Biochemical confirmation was not used to assess alcohol consumption.

2) *Demographics and substance use*. A non-validated baseline questionnaire was provided to participants to assess demographic characteristics and substance use history. In addition to demographic information, this questionnaire included questions such as “How many years have you smoked cigarettes?” and “In the last seven days, how many days did you drink alcohol?”

3) *Intervention completion*. Completion of either intervention was defined via attendance record as attendance at the Quit Day Retreat. This criterion was used because it provided confirmation that a quit attempt was made and because it reflected overall class attendance. For example, all participants who attended four or more classes also attended the Quit Day Retreat, and all who attended one or fewer classes did not attend the Quit Day Retreat”

4) *Practice compliance*. Compliance with daily meditation practice was assessed via telephone calls made to participants, during which they were asked how many minutes they had practiced meditation in the past 24 hours. Calls were made once a day for 5 days prior to the beginning of treatment and once a day for 5 days after the quit day. “Minutes meditated” was averaged across the five calls in each time period, yielding an average number of minutes meditated per day.

5) *Nicotine dependence*. The Fagerstrom Test for Nicotine Dependence (FTND) [65] was administered at baseline to assess nicotine dependence. The FTND is a widely used six-item measure designed to determine the extent to which an individual has developed dependence on nicotine. The measure possesses fair internal consistency (α = .61) and correlates well with biological indices of heaviness of smoking [65]. Example questions include “How soon after you wake up do you smoke your first cigarette?” and “How many cigarettes do you smoke per day?”

6) *Smoking motives*. Motivation for smoking was assessed via the Brief Wisconsin Inventory of Smoking Dependence Motives (WISDM) completed at baseline [66]. The Brief WISDM is a 37-item subset of the original 68-item WISDM questionnaire, loads onto 11 motivational subscales, and has acceptable internal consistency (α = .69). The instrument provides items such as, “Smoking really helps me feel better if I’ve been feeling down,” and uses a 7-point Likert scale ranging from 1 = “not at all true of me” to 7 = “extremely true of me” [66].

7) *Mindfulness*. To assess mindfulness, the Frieburg Mindfulness Inventory (FMI) [67] was administered at the baseline and post-quit assessment visits to assess the potential acquisition of mindfulness skills as a result of the mindfulness or control intervention. Example items include “I pay attention to what’s behind my actions” and “I am open to the experience of the present moment,” and use a 4-point Likert scale with 1 = “rarely” and 4 = “almost always” as possible responses. The shorter 14-item FMI was used and has demonstrated acceptable internal consistency (α = .86) [67].

8) *Distress tolerance*. The Distress Tolerance Scale (DTS) was administered at baseline and two-week post-quit assessment visit to assess how participants tolerated, appraised, absorbed and regulated their distress [68]. The DTS is a 14-item questionnaire, with acceptable internal consistency (α = .89), includes items such as “I can’t handle feeling distressed or upset,” with possible responses on a 5 point Likert scale ranging from 1 = “strongly agree” to 5 = “strongly disagree” [68].

9) *Stress*. The Perceived Stress Scale-10 (PSS) was provided to participants at baseline and at the two-week post-quit assessment visit [69]. The PSS is designed to assess affective reactions to stressors; it is a 10-item questionnaire, and demonstrates acceptable internal consistency (α = .76). The PSS provides questions such as “In the last month, how often have you felt that you were unable to control the important things in your life?” and allows for responses on a 5-point Likert scale with 0 = “never” and 4 = “very often” [69].

10) *Course Acceptability*: A non-validated course evaluation was administered at the 2-week post-quit assessment visit. The instrument included questions for the purpose of assessing use of materials, adherence to practices, and accessibility of various instructions. An example item was “Meditation/Walking has helped me manage cravings” and provided a 7-point Likert scale response from 1 = “completely disagree” to 7 = “completely agree”. The instrument also included four written-answer questions to obtain more open-ended feedback on what was most or least helpful in the interventions.

### Procedure

MTS and ILS interventions lasted six weeks with identical schedules consisting of six 2-hour weekly classes plus a 7-hour Quit Day Retreat on the weekend between classes four and five. Each class was comprised of 45 minutes of instruction, 45 minutes of group discussion, and 30 minutes of meditation (MTS), or silent, non-directed walking (ILS) (Table 1). MTS participants were asked to practice 30 minutes of meditation per day with a guided meditation CD, whereas ILS participants were asked to practice non-directed walking (walking silently and alone without music or a goal destination) for 30 minutes each day. Instructors for both MTS and ILS held Master’s degrees in psychology and had equivalent experience with smoking cessation interventions. Participants in each group received a 50-page manual containing the core material in the intervention written to approximately a ninth grade reading level. Neither MTS nor ILS treatments provided instruction targeted to alcohol abuse. At the Quit Day Retreat, participants in each group were asked to attempt smoking cessation and stop drinking alcohol for one month. Participants were provided with no medications, and the use of smoking or alcohol cessation medications was discouraged. The Quit Day Retreat provided seven hours of instructor-guided mindfulness practice for MTS participants and seven hours of instructor-guided non-directed walking and group interaction for ILS. After the Quit Day Retreat, participants were asked to attend two additional classes, which provided no additional skills training, but provided a forum for group interaction and discussion.

**Table 1:** MTS and ILS participant activities

**Class**	**MTS**	**ILS**
1	Mindfulness Meditation, Body Scan	Stages of Change, Motivation to Quit
2	Mindfulness and Emotions	Physical + Psychological Addiction, Your Addiction
3	Mindfulness and Smoking Triggers	Smoking and the Body, Managing Triggers
4	Mindfulness for Urges	Preparing to Quit–Strategies for Urges
5	Quit Day–Mindfulness Practices	Quit Day–Non–Directed Walking
6	Mindfulness and Withdrawal	Managing Withdrawal
7	Long Term Meditation Practice	Relapse Prevention
	2-week post-quit assessment visit	2-week post-quit assessment visit

The Quit Day Retreat was a 7-hour silent retreat with instructor-guided mindfulness practices.

Each class incorporated a 30-minute guided meditation and 45 minutes of support group interaction as “mindful talking and listening”.

### Control intervention

The Interactive Learning for Smokers (ILS) intervention combined elements of the American Lung Association’s Freedom from Smoking program [70] and The Mayo Clinic’s Nicotine Dependence Center program [71] and was constructed to help participants develop individualized smoking cessation strategies to manage relapse challenges related to smoking triggers, social situations, strong emotions, stressful situations, relapse-related thoughts, urges and withdrawal symptoms–areas also targeted in MTS. ILS participants were asked to practice thirty minutes of silent non-directed walking per day throughout the intervention, and to further match MTS, were instructed to use non-directed walking for relaxation, stress reduction and as a strategy for managing urges and withdrawal symptoms.

### Data analysis

Management of missing data followed recommended methods [72]. Assessment of the primary abstinence and alcohol outcome variables included intent-to-treat analysis as well as analysis of intervention completers. For intent-to-treat analyses, failure to attend the assessment visits resulted in coding the participant as relapsed with return to pre-intervention alcohol use or tobacco use (i.e., missing = smoking). Independent groups *t*-tests and chi-square tests were conducted to compare baseline characteristics between groups. Logistic regression was used to evaluate the treatment group effect on abstinence and to compute odds ratios (OR) estimates and confidence intervals. ANOVAs were used to evaluate repeated measures (continuous variables) over time. Pearson correlations were computed to identify associations between secondary outcome measures and continuous smoking outcomes. Analyses were performed using SPSS, Version XX.

## Results

Participant mean age was 21.9 years (SD = 2.53), 70.9% male, and 83% white (Table 2). Baseline testing showed no statistically significant differences between MTS and ILS groups on gender, race, ethnicity, age, number of cigarettes smoked per day, or average number of drinks per day.

**Table 2:** Participant baseline characteristics

	**Total (SD)**	**MTS**	**ILS**	**p-value**
**Baseline characteristics**	55			
Gender				
Male	70.9%	70.0%	72.0%	.87
Female	29.1%	30.0%	18.0%	
Race				
American Indian	1.8%	3.3%	0.0%	.37
Asian	0.0%	0.0%	0.0%	N/A
African American	1.8%	0.0%	4.0%	.33
Latino/Hispanic*	5.5%	6.7%	4.0%	.67
White	90.9%	90.0%	92.0%	.82
Other	0.0%	0.0%	0.0%	N/A
Age	21.93 (2.53)	21.70 (2.42)	22.20 (2.68)	.47
Number of cigarettes/day	13.75 (6.36)	13.63 (3.90)	13.88 (8.52)	.89
Number of drinks per week**	11.76 (8.36)	12.73 (9.92)	10.30 (5.42)	.49

* Recruitment materials were in English and did not target non-English speaking populations.

** Data collected from completers only from course evaluations (n = 25).

Intent-to-treat chi-square analysis showed that biochemically confirmed 7-day point prevalence smoking abstinence rates at 2-weeks was higher for MTS than ILS, but the finding was not significant (*p* = .08) (Table 3). Similarly, intervention completers demonstrated higher 7-day point prevalent abstinence rates in MTS compared to ILS, but the finding was not significant (*p* = .10). Independent *t*-test showed that MTS had a significantly greater number of days of smoking abstinence in the first two weeks compared to ILS in both intent-to-treat analysis and analysis of completers.

**Table 3:** Smoking outcomes

**Analysis**	***MTS (SD)***	***ILS (SD)***	**χ**^**2**^	***t***	**β**	***Odds Ratio (CI)***	***Wald***	***p-value***
Intent-to-Treat (n = 55) 2-week point prevalence abstinence	20.0% (.41)	4.0% (.20)	3.14	N/A	1.79	6.00 (.67-53.68)	2.57	.08
Completers (n = 25) 2-week point prevalence abstinence	40.0% (.51)	10.0% (.32)	2.68	N/A	1.79	6.00 (.60-60.44)	2.31	.10
Intent-to-Treat (n = 55) Days abstinent two-weeks post-quit	5.10 (6.00)	2.04 (3.98)	N/A	2.26	N/A	N/A	N/A	.03*
Completers (n = 25) Days abstinent two-weeks post-quit	10.20 (4.36)	5.10 (5.00)	N/A	2.71	N/A	N/A	N/A	.01*

*statistically significant.

χ^2^ *- chi-square statistic,* t *- independent samples t-test statistic,* β - Beta coefficient, *CI* - confidence interval.

For those who did not attend follow-up in intent-to-treat analysis, participants were recorded as smoking every day.

Completer post-quit analysis using independent *t*-test showed no significant differences between number of drinks per week in MTS (M = 10.70, SD = 11.94) and ILS (M = 15.25, SD = 8.08), *t*(23) = 1.05, *p* = .30. Completer analysis showed that ILS significantly increased drinks per week from pre-quit (M = 10.30, SD = 5.42) to post-quit (M = 15.25, SD = 8.08), *t*(9) = −2.60, *p* = .03, whereas MTS decreased drinks per week from pre-quit (M = 12.73, SD = 9.92) to post-quit (M = 10.70, SD = 11.94), although the difference was not significant, *t*(14) = .59, *p* = .56. There was not a significant interaction between treatment groups and drinks per week over time, *F*(1, 23) = 2.41, *p* = .14.

Analyses were conducted to determine whether post-quit alcohol consumption was correlated with smoking relapse. Findings showed that among intervention completers, participants who had maintained smoking absti nence at two-weeks post-quit (*n* = 7) reported significantly fewer days drinking alcohol (M = 2.43, SD = 1.90) than participants who were smoking relapsed (*n* = 18) (M = 4.72, SD = 2.67), *t*(23) = 2.06, *p* = .05. Total number of drinks per week post-quit showed a similar pattern, with smoking abstinent participants drinking only 5.0 (SD = 6.64) drinks per week vs. smoking relapsed participants showing 15.44 (SD = 10.56) drinks per week, *t*(23) = 2.42, *p* = 0.02). Number of drinks per week post-quit was significantly negatively correlated with smoking relapse, *r*(25) = −.38, *p* < .05.

Attrition for all participants from randomization to assessment visit was 55% with no significant difference between groups (MTS = 50.0%, ILS Controls = 60.0%), χ^2^(1, *N* = 55) = 0.55, *p* = .46. We found no predictors for attrition, including age, gender, FTND, FMI, DTS, or PSS or WISDM. When comparing those who attended the two-week post-quit assessment visit (completers) to those who did not (non-completers) on baseline variables, we found no significant differences between intervention randomization, gender, ethnicity, age, FTND, FMI, DTS, PSS, or WISDM (Table 4), but did find that completers reported smoking fewer cigarettes per day at baseline (M = 11.88, SD = 3.00) than non-completers (M = 15.30, SD = 7.89), *t*(53) = 2.19, *p* = .04. Participants who had dropped out or missed classes showed that technical college exams and vacations accounted for the majority of attrition. There was no significant difference in class attendance for intervention completers: 80.3% for MTS and 75.0% for ILS, *p* = .44. Mean minutes per day meditating/walking was assessed in course completers and showed means for MTS of 24.41 minutes meditation, and ILS of 46.81 minutes walking (30 min/day requested for each), *p* = .17. Acceptability of the manual was mixed in that almost all completers claimed to have read sections of the manual but only two participants in ILS and three in MTS claimed to have read the entire manual. The primary reason cited for not reading the manual was that most participants did not commonly read books voluntarily.

**Table 4:** Completer vs. non-completer baseline comparison

	**Total (SD)**	**Attended follow-up (Completers)**	**Did not attend follow-up**	**p-value**
**Baseline characteristics**	55	25	30	
Intervention				
MTS	54.5%	60.0%	50.0%	.32
ILS	45.5%	40.0%		
Gender			50.0%	.56
Male	70.9%	72.0%	70.0%	
Female	29.1%	28.0%	30.0%	
Race				
American Indian	1.8%	4.0%	0.0%	.46
Asian	0.0%	0.0%	0.0%	
African American	1.8%	0.0%	3.3%	.55
Latino*	5.5%	8.0%	3.3%	.43
White	90.9%	88.0%	93.4%	.64
Other	0.0%	0.0%	0.0%	
Ethnicity (Hispanic)	5.5%	8.0%	3.3%	.43
Age	21.93 (2.53)	22.00 (2.33)	21.87 (2.73)	.85
Number of cigarettes/day	13.75 (6.36)	11.88 (3.00)	15.30 (7.89)	.04*
FTND	3.25 (1.70)	3.39 (1.59)	3.14 (1.80)	.61
FMI	27.71 (6.31)	29.43 (5.98)	26.29 (6.32)	.08
DTS	3.46 (.90)	3.55 (.90)	3.38 (.91)	.51
PSS	17.78 (7.02)	15.91 (6.13)	19.32 (7.43)	.08
WISDM	53.26 (9.31)	53.06 (7.94)	53.42 (10.44)	.89

*Statistically significant.

**Recruitment materials were in English only.

## Discussion

This pilot study was designed to investigate the potential usefulness of a mindfulness-based therapy in young adult smokers with binge alcohol use. This is of particular interest because of the high prevalence of alcohol use in this age group and the strong relationship between alcohol use and smoking relapse. The study was designed to compare a mindfulness-based intervention to a closely-matched active control. There was no therapy targeted to alcohol use provided to either group, and it was hoped that mindfulness training might have generalized effects on alcohol use. The population was primarily young males in their twenties who engaged in regular heavy binge drinking. Major findings from the study were the following: 1) Smoking abstinence: In our primary outcome, point prevalent smoking abstinence was higher in MTS than controls, but the difference was not significant. As a secondary outcome, MTS compared to ILS participants showed significantly greater number of days abstinent in the first two weeks. 2) Alcohol use: Controls significantly increased alcohol consumption over the course of their intervention whereas MTS participants decreased consumption, but not significantly. 3) Alcohol use and smoking relapse: Post-quit alcohol use was significantly associated with smoking relapse in every measure obtained; 4) Attrition: Attrition was high for both groups; and 5) Acceptability: Class attendance was reasonable for completers; minutes meditated and minutes walked were also reasonably high for completers.

Although this was a pilot study and was underpowered to provide statistically significant differences on many outcomes, the study still yielded some meaningful findings. It is noteworthy that ILS yielded such low smoking cessation rates (4%) although it was an intense intervention, with skilled instructors, group support, skills training, and cognitive behavioral strategies used in evidence-based programs. These low rates make a compelling case that the sample population is an intrinsically challenging group. The modestly higher smoking cessation rates for the MTS intervention (10%) are only noteworthy within the context of understanding the challenges of treating this population. It was further encouraging that number of days smoked in the first two weeks was in fact significantly less for MTS than ILS. The fact that mindfulness training made any difference at all in these densely-layered intensive interventions [73] is surprising, and suggests the possibility that mindfulness training may have had an independent therapeutic effect on smoking.

Alcohol outcomes need to be understood in the context of the fact that this population was young, mostly male, drank heavily, and that neither intervention provided alcohol-specific treatment. The hope was that skills learned from MTS might have generalized to reduce alcohol use. Surprisingly, this study showed that controls significantly increased their alcohol use after the quit day, whereas MTS participants showed a decrease that was non-significant. A possible explanation of this data is that smoking cessation is stressful and might lead to a greater tendency to drink (as seen in ILS), and that mindfulness training attenuated this tendency to drink more post-quit. The findings of this pilot study were not powered sufficiently to demonstrate that mindfulness is an effective simultaneous treatment of comorbid alcohol and tobacco abuse. Given, however, the growing support for simultaneous treatment of both alcohol and tobacco [42-44,46,74,75], and the fact there are already mindfulness-based therapies for the treatment of alcohol [54,55] and tobacco [57,73], the findings suggest that there may be promise for larger studies on mindfulness training for both alcohol and tobacco use.

An expected finding in this study was the association between alcohol use and smoking relapse. Participants who were smoking abstinent compared to relapsed drank significantly fewer days post-quit and showed a significant correlation between abstinence and alcohol use. This is consistent with studies on older populations that show that binge drinking [40] and moderate drinking [41] are correlated with smoking relapse. The finding of a significant correlation in a study with small sample size suggests that the strong relationship between alcohol use and smoking relapse also exists in the young adult population. Findings suggest that it might be beneficial for investigators to further study the relationship of alcohol and tobacco in the young adult population.

Participants who completed the intervention provided relatively positive comments about both MTS and ILS. Among course evaluations, both groups cited group support and instructor support as most important, and each group mentioned meditation (or non-directed walking) as helpful to them. Written reports about the experience of meditation were very positive and suggested that these individuals, though young and prone to high-risk behavior, were also capable of quiet reflection. The use of the manuals was disappointing in both groups, with most participants reading only small portions. When asked why they did not read the manuals, the most common answer was that participants did not normally engage in voluntary reading. This suggests that intervention information might be better provided in another form such as video.

A major limitation in this study was high attrition in every phase–recruitment, retention and follow-up. High attrition rates were first noted during recruitment when only 34% of callers scheduled for an orientation actually attended an orientation. This suggests that this population had high non-compliance before initiation of any intervention. Intervention attrition rates were also similarly high in both groups.

One possible conclusion regarding attrition in this study is that young adult smokers are not very interested in intensive therapies such as those offered. Previous studies on young adult smoker have also shown high attrition in study protocols and poor adherence to behavioral therapies [28,29]. This may be true for many such individuals; however, reports from those who completed the intervention were very positive, suggesting that for a portion of participants the interventions showed good acceptability. It is likely that the completer analyses is reporting results on those participants who were most receptive to mindfulness training, artificially increasing effect sizes. In the future trials on young adult binge drinkers, it would be wise to incorporate design elements to decrease attrition. Such methods might include identification of individuals that are most prone to stay in an intensive intervention, or implementation of incentives for treatment adherence. Another limitation of this study was small sample size. As a pilot study, the sample size was such that even if respectable differences were found between groups, smoking and alcohol outcomes would likely be underpowered to reach significance. A final study limitation is that outcomes were assessed only at 2-weeks post-quit and, as such, the magnitude and pattern of treatment group differences at long-term follow-up (e.g., 6-months post-quit) are unknown.

## Conclusions

This study was designed as part of a mindfulness intervention development effort consistent with NIH-endorsed stage-1 development protocols for testing emerging therapies. Goals of this type of pilot study were to provide understandings of program acceptability, estimates of effect sizes, and insights to guide program development [58]. Primary findings from this study suggest that MTS, compared to a closely matched control, produced non-significant increases in short-term (2-week) smoking abstinence and showed reasonable acceptability among those who completed the intervention. The study also showed non-significant differences in alcohol use in MTS compared to controls. This suggests some promise that mindfulness skills, taught for the purpose of smoking cessation, may generalize to decrease alcohol use. It is hoped that these findings might be useful in the design of future studies that evaluate mindfulness-based treatments for smokers.

## Competing interests

All authors declare that they have no competing interests.

## Authors’ contributions

JD conceived the study, obtained funding for the study, oversaw implementation of the study, and wrote the manuscript; DM assisted in data analysis and manuscript preparation; KS assisted in background research, data cleaning and manuscript preparation; AM assisted in data analysis and manuscript preparation; MM assisted in original study design, teaching the intervention and manuscript editing; SS assisted in data analysis and manuscript preparation. All authors read and approved the final manuscript.

## Pre-publication history

The pre-publication history for this paper can be accessed here:

http://www.biomedcentral.com/1472-6882/13/215/prepub
